# From incivility to aggression: latent profiles of outsider mistreatment and their association with well-being

**DOI:** 10.1108/JHOM-09-2025-0602

**Published:** 2026-04-21

**Authors:** Elena Cavallari, Valentina Sommovigo, Ilaria Setti

**Affiliations:** Department of Brain and Behavioral Sciences, University of Pavia, Pavia, Italy; Department of Economics and Management, University of Pavia, Pavia, Italy

**Keywords:** Healthcare professionals, Aggression, Incivility, Latent profile analysis, Person-centred approach, Occupational well-being

## Abstract

**Purpose:**

Outsider mistreatment—aggressive or disrespectful behaviours by patients, relatives, or visitors—poses a critical threat to healthcare workers' well-being and organisational functioning. Guided by the Conservation of Resources theory, this person-centred study examined exposure patterns and their associations with psychological and occupational outcomes among healthcare professionals.

**Design/methodology/approach:**

Responses from 2,219 healthcare professionals in eight Northern Italian hospitals were analysed using latent profile analysis to identify subgroups with varying levels of exposure to mistreatment.

**Findings:**

Two distinct profiles emerged: Low Mistreatment Exposure (89.1%) and Moderate–High Mistreatment Exposure (10.9%). The latter was marked by elevated incivility, verbal aggression, and especially physical aggression, and was associated with greater emotional exhaustion, mental distancing, psychosomatic symptoms, and post-traumatic stress symptoms, alongside lower job satisfaction, reduced trauma-related coping self-efficacy (CSE), and stronger turnover intentions. These findings highlight the cumulative and multifaceted nature of outsider mistreatment and its uneven impact across staff.

**Originality/value:**

This study represents the first application of a person-centred approach latent profile analysis, specifically to outsider mistreatment in the healthcare sector. By integrating incivility and aggression into a unified continuum, the research moves beyond traditional variable-centred analyses. Moreover, it extends existing research by linking these profiles to both occupational well-being and CSE, highlighting differential pathways of vulnerability and resilience.

## 1. Introduction

Healthcare professionals work in high-pressure settings. However, their work is often met with disrespect and aggression. Globally, 8–38% report being physically attacked during their careers (World Health Organization, 2021). Many more experience verbal threats or abuse, mainly from patients or relatives (Mento *et al.*, 2020; Yusoff *et al.*, 2023). These incidents have increased since the onset of COVID-19, highlighting the need for improved prevention and support (Oerther *et al.*, 2021; The World Medical Association, 2022).

Healthcare workers often face inappropriate and hostile acts from patients, relatives, and visitors (Ma and Thomas, 2025; Yusoff *et al.*, 2023). These acts typically manifest as incivility or verbal or physical aggression (NIOSH, 2022). Aggression involves a clear intent to harm (e.g. pushing, hitting, threats, insults) (Cavallari *et al.*, 2024; NIOSH, 2022). In contrast, incivility refers to low-intensity acts that violate norms and are characterised by ambiguous intent to harm (e.g. ignoring, dismissive gestures, demeaning comments) (Andersson and Pearson, 1999; Cortina *et al.*, 2017; Townsley *et al.*, 2023). Although often studied separately (Zappalà *et al.*, 2021), theoretical and empirical work increasingly suggests that these behaviours are interrelated and may co-occur along a continuum of mistreatment (Townsley *et al.*, 2023). The continuum varies in severity, frequency, intentionality, and subjective appraisal (Hershcovis, 2011; NIOSH, 2022; Townsley *et al.*, 2023). Repeated low-intensity acts can have cumulative effects comparable to those of a single severe incident (Chris *et al.*, 2022; Sommovigo *et al.*, 2024). If ignored, even minor acts may escalate into overt verbal or physical aggression—known as the incivility spiral (Andersson and Pearson, 1999; Thomas *et al.*, 2022). Accordingly, outsider mistreatment —defined as disrespectful or aggressive behaviour from individuals external to the organisation (Bowling and Beehr, 2006)— is best understood not as a series of isolated events but as a cumulative and potentially escalating occupational stressor (Cavallari *et al*., 2025b; Townsley *et al.*, 2023).

To clarify the theoretical lens, the present study draws on the Conservation of Resources (COR) theory (Hobfoll *et al.*, 2018), a central framework in occupational stress research. COR theory holds that stress arises when valued resources are threatened or lost and that resource loss outweighs gains (Cavallari *et al.*, 2025a). This dynamic that can accumulate over time as loss spirals (Hobfoll *et al.*, 2018). Outsider mistreatment serves as a resource-depleting stressor within this framework, providing a direct theoretical basis for examining its effects on healthcare professionals. Empirical research consistently links outsider mistreatment to adverse individual and organisational outcomes. These include emotional exhaustion, psychological distress, reduced job satisfaction, and turnover intentions (Mawuena *et al.*, 2024; Mento *et al.*, 2020; O'Brien *et al.*, 2024; Wang *et al.*, 2022; Zheng *et al.*, 2022). In COR terms, repeated mistreatment depletes vital resources (e.g. emotional stability, self-efficacy, social support) (Hobfoll *et al.*, 2018; Sommovigo *et al.*, 2024). It also constrains opportunities for resource replenishment, raising vulnerability to stress-related impairment (Cavallari *et al*., 2025b; Hobfoll *et al.*, 2018). When exposure is severe, outsider mistreatment may function as a potentially traumatic occupational stressor. It can elicit post-traumatic stress reactions (Hobfoll *et al.*, 2018; Lee *et al.*, 2020; Wagner *et al.*, 2024). Within this process, trauma-related coping self-efficacy (CSE)—defined as beliefs in one's capacity to regulate distress, manage trauma-related demands, and sustain functioning under threat (Benight and Bandura, 2004)—is a core personal resource within COR terms. High CSE supports adaptive coping and recovery following adversity (Benight *et al.*, 2015; Bosmans and van der Velden, 2017; Sommovigo *et al.*, 2025a). Resource loss can progressively undermine these beliefs, weakening individuals' capacity to cope with later stressors (Benight *et al.*, 2015; Luszczynska *et al.*, 2009; Sommovigo *et al.*, 2025a). Importantly, from a COR perspective, such erosion is not uniform across individuals. Instead, it varies with exposure intensity, the availability of personal resources, and the surrounding organisational resource environment (Hobfoll *et al.*, 2018). These factors jointly shape differential vulnerability to loss spirals. Exposure and impact also vary with individual characteristics (e.g. gender, role, tenure) and organisational conditions (e.g. workload, safety protocols, support availability) (Cavallari *et al.*, 2025a; Ellis *et al.*, 2019; Lim *et al.*, 2022). Nevertheless, most research has relied on variable-centred approaches that assume homogeneous exposure-outcome relationships. This conceals critical heterogeneity in how outsider mistreatment is perceived and processed (Gabriel *et al.*, 2018; Howard and Hoffman, 2018).

Despite the prevalence and severity of outsider mistreatment in healthcare, the literature has three key limitations. First, most studies use variable-centred approaches that assume population homogeneity. This obscures the fact that healthcare professionals experience different patterns of mistreatment. These patterns have unequal effects on resource loss and adjustment, as COR theory predicts. Second, person-centred studies are emerging, but they focus mainly on internal mistreatment sources such as bullying or lateral violence. This leaves the diversity of outsider-initiated aggression largely unexplored. Third, little is known about how different patterns of exposure to mistreatment affect CSE. CSE is a core personal resource for recovery after potentially traumatic work events. To address these gaps, this study uses person-centred latent profile analysis (LPA) to examine patterns of outsider mistreatment. The sample includes 2,219 healthcare professionals from eight public hospitals, a context rarely examined with person-centred research. Guided by COR theory, this approach characterizes mistreatment not as uniform but as differentiated exposure profiles, each linked to distinct resource-loss dynamics. The study makes three related contributions. Methodologically, it identifies qualitatively distinct exposure profiles, showing that outsider mistreatment is not uniform but organised into discrete configurations. This challenges the one-size-fits-all assumptions of dominant stress models. Theoretically, it extends COR theory by showing that chronic outsider aggression forms differentiated profiles, with higher-risk profiles linked to cumulative resource loss. These losses are associated with poorer psychological well-being, reduced work functioning, and the loss of coping resources, especially CSE. This amplifies vulnerability through loss spirals. Conceptually, integrating incivility, verbal, and physical aggression in a single person-centred defines outsider mistreatment as a cumulative, potentially traumatic occupational phenomenon, rather than isolated incidents. Practically, the findings provide a data-driven basis for stratified interventions. These shifts focus from uniform approaches to targeted, subgroup-specific strategies. By linking research on mistreatment and COR-based resource dynamics, the study offers a fresh view of how outsider mistreatment operates as a diverse, resource-draining issue within public healthcare.

### 1.1 Latent profile analysis in outsider mistreatment literature

Building on the preceding COR-based argument, outsider mistreatment acts as a cumulative and heterogeneous stressor. LPA is a person-centred approach that adopts a holistic perspective. It examines how multiple variables co-occur within individuals, forming distinct patterns of behaviour and psychology (Gabriel *et al.*, 2018; Sommovigo *et al.*, 2025b). Variable-centred approaches focus on average associations across populations and assume homogeneity of exposure and effect. In contrast, LPA identifies latent subgroups characterised by distinct configurations of variables, either qualitatively or quantitatively (Sommovigo *et al.*, 2025b). This methodological distinction is relevant from a COR perspective. COR theory holds that stressors and resources are unevenly distributed (Hobfoll *et al.*, 2018). Resource loss processes are expected to be heterogeneous. Individuals differ in exposure intensity, initial resources, and access to contextual support. As a result, stressors such as outsider mistreatment are unlikely to affect the workforce uniformly. Instead, they are expected to produce distinct patterns of cumulative resource loss and adaptation. LPA is well-suited to operationalising this COR-based assumption. It identifies subgroups that experience systematically different demands and resource erosion.

Although increasingly used in organisational research (Moreno-Fernández *et al.*, 2020; Vaziri *et al.*, 2020), LPA has rarely examined outsider mistreatment in healthcare. Prior studies have modelled profiles based on psychological resources or stress outcomes, treating mistreatment as a contextual factor (Chen *et al.*, 2024; Nguyen and Stinglhamber, 2020). Others have focused on internal sources of mistreatment, such as bullying or lateral violence (Baek and Trinkoff, 2022; Chan *et al.*, 2022; Namin *et al.*, 2022; Pina *et al.*, 2022). No research has yet examined whether public hospital healthcare workers form distinct latent profiles based on direct exposure to incivility, verbal, and physical aggression from outsiders. This is a critical gap. Outsider mistreatment is cumulative, multifaceted, and embedded in structurally asymmetric interactions with patients, relatives, and visitors (NIOSH, 2022). It is unlikely that all healthcare professionals experience it uniformly. Some may face repeated and severe hostility. Others may encounter only sporadic or low-intensity episodes. From a COR perspective, outsider mistreatment should be seen as a stressor that clusters into distinct exposure types, each with differing effects on resource loss.

A person-centred approach, such as LPA, captures this theoretical idea directly. It enables identification of subgroups with shared exposure patterns and vulnerability to resource loss. Recognising these profiles is essential for understanding different vulnerabilities and forms of resilience. It is also vital for designing targeted interventions for specific segments of the healthcare workforce. Thus, in line with this COR-based reasoning, we hypothesise.

H1.
Distinct latent profiles will emerge based on healthcare workers' perceived exposure to mistreatment by outsiders.

### 1.2 Outsider mistreatment and its outcomes

Outsider mistreatment—manifested through disrespectful or aggressive behaviours by patients, relatives, or visitors—represents a salient occupational stressor in healthcare, threatening both individual well-being and organisational functioning (Cavallari *et al*., 2025b; Sommovigo *et al.*, 2022). In line with the COR theory (Hobfoll *et al*., 2018), outsider mistreatment can be conceptualised as a stressor that initiates processes of resource loss. Specifically, emotionally demanding efforts (e.g. empathic communication) may fail to yield expected gains (e.g. recognition), while also eroding personal resources (e.g. emotional stability) and valued social-organisational conditions (e.g. trust and safety at work) (Cavallari *et al.*, 2025a; Sommovigo *et al.*, 2022). Extant research consistently links such cumulative resource depletion to key indicators of occupational strain, including emotional exhaustion, the central component of burnout characterised by chronic physical and emotional fatigue (Sommovigo *et al.*, 2025a; Sommovigo *et al.*, 2022). As resource losses accumulate, employees may experience psychosomatic strain (e.g. sleep disturbances, headaches, gastrointestinal problems) (Mento *et al.*, 2020; Zhang *et al.*, 2024) and adopt withdrawal strategies, such as mental distancing (i.e. psychological detachment or cynicism from one's work (Cavallari *et al.*, 2025a; Consiglio *et al.*, 2021), which COR theory conceptualises as defensive responses aimed at conserving remaining resources (Schaufeli *et al.*, 2023). Although such strategies may buffer short-term strain, they are associated with lower job satisfaction (i.e. the extent to which individuals feel positive and fulfilled by their work) and stronger turnover intentions (i.e. the conscious motivation to leave one's job or organisation), thereby undermining organisational stability and care quality (Ajaiyeoba and Aplin-Houtz, 2025; Cavallari *et al.*, 2025a; Gabriel *et al.*, 2018; Lim *et al.*, 2022). Importantly, COR theory also provides a framework for understanding why the consequences of outsider mistreatment may extend beyond strain to trauma-related outcomes. In severe cases, outsider mistreatment may act as a traumatic stressor, eliciting post-traumatic stress symptoms (PTSSs) such as intrusion (i.e. the involuntary re-experiencing of aggressive events through distressing memories, flashbacks, or nightmares), avoidance (i.e. the tendency to disengage from trauma-related reminders, thoughts, or interactions), and hyperarousal (i.e. a state of heightened physiological vigilance manifested in irritability, concentration difficulties, sleep disturbances, and a constant sense of being “on guard”) (Dai *et al.*, 2024; Montani *et al.*, 2020; Sommovigo *et al.*, 2025a).

However, COR theory also emphasises that individuals' vulnerability to stressors depends on the availability and strength of their personal resources (Hobfoll *et al.*, 2018). In this regard, CSE is a key personal resource. CSE encompasses confidence in regulating negative emotions, confronting trauma-related reminders, and persisting in coping efforts despite distress (Bosmans and van der Velden, 2017; Sommovigo *et al.*, 2025a). Employees with higher CSE tend to perceive stressors as less threatening and report fewer PTSSs over time (Bosmans *et al.*, 2016; Luszczynska *et al.*, 2009). However, COR theory further highlights that repeated or intense losses may erode even this core coping resource, leading to diminished CSE and more severe impairments in well-being and work functioning (Setti *et al.*, 2018; Sommovigo *et al.*, 2018). Accordingly, the consequences of mistreatment are expected to vary across healthcare professionals as a function of their exposure configuration. Such exposure patterns—varying in frequency, intensity, and perceived severity—are expected to produce differentiated patterns of resource loss and adjustment. Employees exposed to chronic and hostile interactions may experience cumulative resource depletion reflected in heightened emotional and occupational strain. In contrast, those facing occasional or low-intensity mistreatment may retain sufficient resources to maintain well-being and engagement.

A person-centred approach, such as LPA, enables the identification of subgroups that share distinct exposure patterns and associated outcomes (Howard and Hoffman, 2018). Prior research supports the value of this approach. Nurses who were bullied reported more medical errors and poorer work–life balance than those who were less exposed (Chan *et al.*, 2022). Those with high violence profiles showed more exhaustion, less job satisfaction, and more somatic symptoms than those with low violence profiles (Pina *et al.*, 2022). Frontline employees who experienced incivility reported greater exhaustion, stronger intentions to quit, and lower performance (Namin *et al.*, 2022).

Taken together, this COR-informed body of research suggests that the consequences of outsider mistreatment depend on the configuration, intensity, and chronic accumulation of exposure, reflecting heterogeneous resource loss processes and loss spirals. This theoretical perspective therefore justifies a person-centred comparison of outcomes across distinct exposure profiles, rather than assuming uniform effects across the workforce.

Consistent with COR theory, we therefore expect high-exposure profiles to show more emotional exhaustion, psychosomatic symptoms, mental distance, and PTSSs. They will also have less job satisfaction and higher turnover intentions. We further expect these profiles to differ in CSE, reflecting differential erosion of coping resources under chronic exposure. Accordingly, we propose the following.

H2.
There will be significant differences in emotional exhaustion, mental distance, psychosomatic symptoms, post-traumatic stress symptoms, CSE, job satisfaction, and turnover intentions across the identified outsider mistreatment profiles.

## 2. Method

### 2.1 Participants and procedure

Between May and September 2024, data were collected from eight hospitals in Northern Italy. Healthcare professionals were selected as the target population because they are routinely exposed to sustained, emotionally charged interactions with users, making them particularly suitable for examining heterogeneous patterns of outsider mistreatment and their implications for occupational well-being. Recruitment followed a top-down organisational procedure consistent with the research–intervention framework. The research team prepared a formal, detailed communication outlining the project's objectives, scope, and operational procedures. This communication was first shared with departmental management and subsequently disseminated to all eligible healthcare professionals through their direct supervisors. Participation was voluntary, and data were collected via an anonymous online survey. A non-probabilistic, voluntary response sampling strategy was adopted, which is common in applied occupational health research and research–intervention contexts, particularly when studying sensitive phenomena such as incivility and aggression. The large sample size and inclusion of multiple hospital settings help mitigate potential selection bias, ensuring broad representation of the region's healthcare workforce. Informed consent was obtained from all participants. The study followed the Italian National Psychological Association's standards and received approval from the Ethical Committee of the University of Pavia (Protocol Code: 175/24). Study information was first shared with departmental management and then with employees via their supervisors. Informed consent was obtained from all participants. The online survey took about 15 min to complete.

An *a priori* power analysis was not conducted, as power estimation in latent profile analysis is complex and typically requires prior knowledge of population parameter values that are often unavailable (Dziak *et al.*, 2014; Tein *et al.*, 2013; Woo *et al.*, 2018). Accordingly, guidance for LPA commonly relies on evidence from prior research and simulation-based rules of thumb. Reviews of person-centred studies report a median sample size of approximately 500, and simulation studies indicate that samples of this size are generally sufficient to accurately identify the correct number of latent profiles (Nylund *et al.*, 2007; Spurk *et al.*, 2020). The final sample size of the present study (*N* = 2,219) substantially exceeds these benchmarks, supporting the robustness and stability of the identified profile solution.

A total of approximately 7,340 healthcare professionals were invited to participate; 2,368 started the survey, and 2,276 valid responses remained after excluding incomplete cases (*n* = 92). An additional 56 multivariate outliers were removed, resulting in a final sample of 2,219 (response rate = 30.23%). Missing data on continuous variables ranged from 0% to 4.4%, and Little's MCAR test indicated randomness (*χ*^2^ = 15.95, df = 13, *p* = 0.25). Most participants were female (76.9%) with a mean age of 47.0 years (SD = 11.25). On average, they spent 75.3% (SD = 23.9) of their working time in direct contact with the public. Nurses were the largest professional group (34.8%), with other roles distributed across departments.

### 2.2 Measurements

*Outsider Incivility* was assessed using the four-item Italian Workplace Incivility Scale (Sommovigo *et al.*, 2024, α = 0.91). Participants reported how often they experienced discourteous customer behaviour (e.g. “Customers made demeaning, rude, or derogatory remarks about me”). Responses were recorded on a five-point scale (1 = Never to 5 = Always).

*Outsider Aggression* was measured using the eight-item Italian Hospital Aggressive Behaviour Scale (Cavallari *et al.*, 2024). This scale captures non-physical (α = 0.88) and physical (α = 0.71) aggression (e.g. “Users have shoved, shaken, or spat at me”). Responses were recorded on a five-point scale (0 = Never to 4 = Daily).

*Burnout* was assessed using the Italian Short Burnout Assessment Tool (Consiglio *et al.*, 2021). This scale covers exhaustion (α = 0.94), mental distance (α = 0.83), and psychosomatic complaints (α = 0.78). An example item is “At work, I feel mentally exhausted.” Items were rated on a five-point scale (1 = Never to 5 = Always).

Post-traumatic stress symptoms (PTSS) were assessed using the six-item Impact of Event Scale-Revised (Setti *et al.*, 2018). This scale measures intrusion, avoidance, and hyperarousal (e.g. “Unwanted thoughts about the event kept recurring”). Participants responded on a four-point scale (1 = Never to 4 = Often). A composite score was computed (α = 0.89).

*Trauma-Related Coping Self-Efficacy* (CSE) was assessed using the six-item Italian Coping Self-Efficacy Scale (Sommovigo *et al.*, 2025a), adapted for healthcare professionals exposed to outsider aggression. Participants rated their perceived ability to manage the psychological and emotional aftermath of user-initiated aggression (e.g. *managing distressing thoughts or memories related to the incident*; α = 0.92). Responses were provided on a seven-point Likert scale (1 = *Completely incapable* to 7 = *Perfectly capable*).

*Job satisfaction* was assessed using a single item: *How satisfied have you been with your work?* (Giorgi *et al.*, 2015). Participants responded on a 10-point scale from 0 (*No satisfaction)* to 10 (*Complete satisfaction*).

*Turnover intentions* were measured with the question: *“I am seriously considering leaving my current organisation”* (Santarpia *et al.*, 2023). Participants answered on a seven-point Likert scale from 1 (*strongly disagree*) to 7 (*strongly agree*).

### 2.3 Statistical analyses

Analyses were conducted using IBM SPSS Statistics 25 and Mplus version 8 (Barrett *et al.*, 2019; Muthén and Muthén, 2012). In SPSS, we screened for multicollinearity and computed bivariate correlations. We then evaluated the measurement model via confirmatory factor analysis (CFA) in Mplus, using maximum-likelihood estimation with robust standard errors (MLR). Given the self-report design, we examined potential common method variance using Harman's single-factor test. To further account for potential method effects, we also tested a CFA that included an unmeasured latent method factor (Podsakoff *et al.*, 2003).

After establishing a satisfactory fit, mistreatment by outsiders was modelled using a bifactor structure. A bifactor approach was adopted because intercorrelations among mistreatment dimensions were moderate to high, indicating the presence of a general mistreatment factor and form-specific variance (Morin *et al.*, 2017). Bifactor scores were then used as input for the LPA. LPAs were estimated using MLR in Mplus 8.2. We used 3,000 random starts and retained the 100 best solutions for final optimisation (Sommovigo *et al.*, 2025b). Model selection prioritised theoretical coherence and interpretability (Sommovigo *et al.*, 2025b). Model fit was assessed using log-likelihood, Akaike's Information Criterion (AIC), Bayesian Information Criterion (BIC), and sample-size–adjusted BIC (SABIC), with lower values indicating a better fit. Profiles representing less than 1% of the total sample (*N*) or fewer than 25 cases were excluded because such solutions are typically unstable and offer limited theoretical and practical interpretability (Spurk *et al.*, 2020). Relative model fit was further evaluated using the Lo–Mendell–Rubin adjusted likelihood ratio test (LMR-LRT), the Vuong–Lo–Mendell–Rubin likelihood ratio test (VLMR), and the bootstrap likelihood ratio test (BLRT). A significant *p*-value indicated that a model with k profiles fitted the data better than a model with k−1 profiles. Classification accuracy was assessed using entropy. Values ≥ 0.60 indicated acceptable separation (Wang and Hanges, 2011). After selecting the optimal solution, we conducted independent-samples *t*-tests. These tests confirmed the distinctiveness of the profile in incivility, verbal aggression, and physical aggression. We also examined between-profile differences in health-related variables, trauma-related CSE, job satisfaction, and turnover intentions.

## 3. Results

### 3.1 Descriptive analyses

First, we assessed multicollinearity by confirming that variance inflation factors (1.15–2.99) and tolerance values (0.33–0.79) were within acceptable ranges, indicating that multicollinearity was not a concern. Next, we examined skewness (−1.06 to 2.92) and kurtosis (−0.36 to 8.43), which indicated departures from normality. Despite this, all items loaded significantly on their intended factors (≥0.53), with satisfactory factor loadings across constructs: incivility (0.75–0.86), verbal aggression (0.63–0.84), physical aggression (0.70–0.80), exhaustion (0.73–0.88), mental distance (0.60–0.82), psychosomatic symptoms (0.63–0.70), PTSS (0.53–0.88), and CSE (0.71–0.85). Reliability indices were robust, with composite reliability ranging from 0.78 to 0.96, AVEs from 0.50 to 0.68, and Cronbach's αs from 0.79 to 0.94. Finally, correlational analyses (Table 1) showed that all forms of outsider mistreatment were positively related to emotional exhaustion, mental distance, psychosomatic complaints, and PTSS, and negatively associated with CSE and job satisfaction.

**Table 1:** Intercorrelations and descriptive statistics among study variables (*N* = 2,219)

	M	SD	CR	AVE	Skew.	Kur.	1	2	3	4	5	6	7	8	9	10	11	12
1. Incivility	0.95	0.95	0.91	0.68	1.06	0.51	*0.91*											
2. VA	0.93	0.88	0.89	0.57	1.12	0.81	0.80^**a^	*0.88*										
3. PA	0.15	0.40	0.72	0.57	2.92	8.43	0.35^**a^	0.34^**a^	*0.71*									
4. Exh	2.50	0.98	0.94	0.66	0.51	−0.34	0.37^**a^	0.40^**a^	0.19^**a^	*0.94*								
5. Dist	1.78	0.79	0.84	0.52	1.29	1.51	0.32^**a^	0.35^**a^	0.14^**a^	0.60^**a^	*0.83*							
6. Psychos	2.15	0.87	0.79	0.50	0.75	−0.00	0.30^**a^	0.30^**a^	0.14^**a^	0.58^**a^	0.43^**a^	*0.78*						
7. PSST	1.07	0.92	0.90	0.60	0.69	−0.21	0.41^**a^	0.42^**a^	0.19^**a^	0.51^**a^	0.45^**a^	0.46^**a^	*0*.*89*					
8. CSE	5.63	1.30	0.92	0.66	−1.09	0.90	−0.18^**a^	−0.22^**a^	−0.07^**a^	−0.32^**a^	−0.36^**a^	−0.27^**a^	−0.38^**a^	*0.92*				
9. Satisf	6.47	2.32	–	–	−0.89	0.36	−0.23^**a^	−0.25^**a^	−0.12^**a^	−0.49^**a^	−0.59^**a^	−0.35^**a^	−0.29^**a^	0.31^**a^	–			
10. Turn.	2.63	1.93	–	–	0.97	−0.36	0.17^**a^	0.21^**a^	0.11^**a^	0.46^**a^	0.50^**a^	0.32^**a^	0.29^**a^	−0.24^**a^	−0.52^**a^	–		
11.Sex	–	–	–	–	–	–	0.12^**b^	0.07^**b^	0.05^**b^	0.08^**b^	−0.01^b^	0.24^**b^	0.06^**b^	−0.04^b^	−0.07^**b^	−0.00^**b^	–	
12. Age	47.01	11.25	–	–	–	–	−0.16^**c^	−0.10^**c^	−0.07^**c^	−0.03^*c^	−0.05^**c^	−0.01^c^	0.01^c^	0.04^*c^	0.04^c^	−0.07^**c^	−0.04^b^	–
13. Contact	75.28	23.94	–	–	–	–	0.15^**c^	0.12^**c^	0.15^**c^	0.05^**c^	0.03^*c^	0.07^**c^	0.06^**c^	−0.01^c^	−0.03^c^	0.02^c^	0.03^b^	−0.10^**b^

**Note(s):** Incivility = outsider incivility; VA = verbal aggression from outsiders; PA = physical aggression from outsiders; Exh. = emotional exhaustion; Dist. = mental distance; Psychos = Psycho-somatic symptoms; PTSS = post-traumatic stress symptoms; CSE = trauma-related coping self-efficacy; Satisf. = Job satisfaction; Turn. = turnover intentions. Sex: biological sex: 0 = male, 1 = female; age in years; direct contact with outsiders = percentage of working time spent in direct contact with the public. M = mean; SD = standard deviation; CR = composite reliability; AVE = average variance extracted; Skew. = skewness; Kur. = kurtosis. Italic values on the diagonal indicate Cronbach's alpha. *p < 05; **p < 0.01. a = Pearson's correlations; b = Spearman's rho; c = Kendall's tau-b. *p < 0.05, **p < 0.01

**Source(s):** Authors' own work

### 3.2 Confirmatory factor analyses

The CFA (Table 2) supported the proposed eight-factor model. This model demonstrated excellent fit (*χ*^2^ = 4151.32, df = 832, *p* < 0.001; CFI = 0.93; TLI = 0.92; RMSEA = 0.04, 90% CI [0.04, 0.04]; SRMR = 0.04). Competing models, including single- and combined-factor solutions, showed significantly poorer fit, confirming the superiority of the eight-factor structure. Harman's single-factor test showed that the first factor accounted for only 31.73% of the variance, and an unmeasured latent method factor accounted for 22.47%. Both values were well below commonly used thresholds in self-report research, indicating that common method variance is unlikely to compromise the validity of the findings.

**Table 2:** Fit indices for the hypothesised CFA model and alternative measurement models

Model	*χ* ^2^	df	*p*	RMSEA	RMSEA [90% CI]	SRMR	CFI	TLI
8-factor model^i^	3703.55	789	0.000	0.04	[0.04, 0.04]	0.03	0.95	0.95
8-factor model^h^	4151.32	832	000	0.04	[0.04,0.04]	0.04	0.93	0.92
7-factor model^g^	4689.10	839	0.00	0.04	[0.04, 0.05]	0.04	0.92	0.91
6-factor model^f^	6396.59	845	0.00	0.05	[0.05, 0.06]	0.05	0.88	0.88
5-factor model^e^	7361.56	850	0.00	0.06	[0.06, 0.06]	0.06	0.86	0.86
4-factor model^d^	7854.41	854	0.00	0.06	[0.06, 0.06]	0.06	0.85	0.85
3-factor model^c^	13139.89	857	0.00	0.08	[0.08,0.08]	0.08	0.74	0.73
2-factor model^b^	17024.48	859	0.00	0.09	[0.09, 0.09]	0.09	0.66	0.64
1-factor model^a^	26153.21	860	0.00	0.11	[0.11, 0.12]	0.12	0.47	0.45

**Note(s):** df = degree of freedom; RMSEA = root mean square error of approximation; SRMR = standardized root mean square residuals; CFI = comparative fit index; TLI = Tucker-Lewis index. Model a = one-factor model; model b = two-factor model in which incivility, verbal aggression, and physical aggression load on the first factor, exhaustion, mental distance, psycho-physical malaise, PTSS, and CSE load on the second factor; model c = three-factor model in which incivility, verbal aggression, and physical aggression load on the first factor, exhaustion, mental distance, psycho-physical malaise load on the second factor, PTTS, and CSE load on the third factor; model d = four-factor model in which incivility, verbal aggression, and physical aggression load on the first factor, exhaustion, mental distance, psycho-physical malaise load on the second factor, PTTS load on the third factor, and CSE loads on the fourth factor; model e = five-factor model in which incivility loads on the first factor, verbal aggression and physical aggression load on the second factor, exhaustion, mental distance, and psycho-physical malaise load on the third factor, PTTS load on the fourth factor, and CSE loads on the fifth factor; model f = six-factor model in which incivility loads on the first factor, verbal aggression, and physical aggression load on the second factor, exhaustion, and mental distance load on the third factor, psycho-physical malaise loads on the fourth factor, PTTS load on the fifth factor, and CSE loads on the sixth factor; model g = seven-factor model in which incivility loads on the first factor, verbal aggression, and physical aggression load on the second factor, mental distance load on the third factor, psycho-physical malaise loads on the fourth factor, exhaustion loads on the fifth factor, PTTS load on the sixth factor, and CSE loads on the seventh factor; model h = eight-factor model in which incivility, verbal aggression, physical aggression, exhaustion, mental distance, psycho-physical malaise, PTTS, and CSE load on different factors; model h = hypothesized eight-factor model; model i = eight-factor model with an additional common method latent factor

**Source(s):** Authors' own work

### 3.3 Latent profile analyses

**Figure 1:**
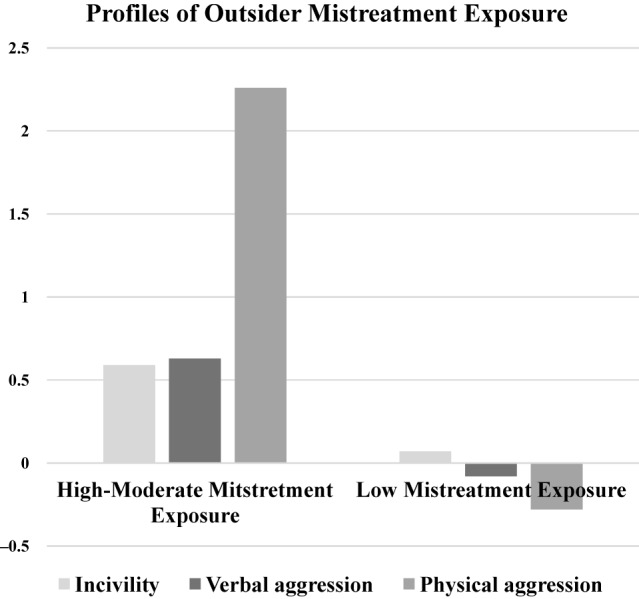
Profile characteristics across indicators (standardised Z scores). **Source:** Authors' own work

Table 3 reports fit indices for LPAs with up to four profiles. The two-profile solution was identified as the most parsimonious and robust, showing clear improvements over the one-profile model in LL, AIC, BIC, and SABIC. Entropy was very high (0.98), indicating excellent classification accuracy. This solution was restrained and evaluated against theoretical expectations and prior research (Chan *et al.*, 2022; Pina *et al.*, 2022). Significant LRT and BLRT results further supported the two-profile solution. Although three- and four-profile models offered marginal statistical improvements, they lacked theoretical justification, and the smallest subgroup in the three-profile model (4.19%) limited interpretability. Therefore, the two-profile model was retained as the most conceptually and empirically sound solution. Subsequently, independent *t*-tests (Table 4) confirmed that professionals in the Moderate-High Mistreatment Exposure profile reported significantly greater exposure to incivility (M = 1.51, SD = 0.99; *t*(2,217) = 9.90, *p* < 0.001), verbal aggression (M = 1.49, SD = 1.01; *t*(2,217) = 10.71, *p* < 0.001), and, especially, physical aggression (M = 1.07, SD = 0.56; *t*(2,217) = 60.95, *p* < 0.001) than those in the Low Mistreatment Exposure profile (M = 0.89, SD = 0.92; M = 0.86, SD = 0.84; M = 0.04, SD = 0.17, respectively). Effect sizes were large for incivility (d = 0.65), verbal aggression (d = 0.68), and physical aggression (d = 2.49), reflecting the near absence of physical aggression in the low-exposure profile. Finally, standardised Z-scores (Figure 1) further delineated the profiles.

**Table 3:** Fit indices from latent profile analyses based on bifactor scores

k	LL	AIC	BIC	SABIC	Entropy	LRT	Adj. LRT *p*	BLRT *p*
1	−5992.98	11997.95	12032.18	12013.12	/	/	/	/
2	−5178.28	10376.56	10433.61	10401.84	0.98	1578.19	0.0029	0.0300
3	−4586.72	9201.43	9281.30	9236.82	0.99	23.76	0.4481	0.000
4	−4349.31	8734.63	8837.31	8780.12	0.99	58.67	0.6450	0.000

**Note(s):** k = number of latent profiles; LL = log-likelihood; AIC = Akaike information criterion; BIC= Bayesian information criterion; SABIC = sample-adjusted BIC; LRT = Vuong-Lo-Mendell-Rubin likelihood ratio test; Adj. LRT *p* = Vuong-Lo-Mendell-Rubin Adjusted Likelihood Ratio Test *p*-value; BLRT *p* = bootstrap likelihood ratio test *p*-value

**Source(s):** Authors' own work

**Table 4:** Means, standard deviations, and group comparisons across mistreatment exposure profiles

	Moderate-high mistreatment exposure (*n* = 243)		Low mistreatment exposure (*n* = 1976)		*t*	*p*	95% CI		Cohen's *d*
	M	SD	M	SD			LL	UL	
Incivility	1.51	0.99	0.89	0.92	9.90	0.000	0.50	0.75	0.65
Verbal aggression	1.49	1.01	0.86	0.84	10.71	0.000	0.51	0.74	0.68
Physical aggression	1.07	0.56	0.04	0.17	60.95	0.000	0.99	1.06	2.49
Emotional exhaustion	2.90	1.00	2.45	0.96	6.86	0.000	0.32	0.58	0.46
Mental distance	1.96	0.86	1.76	0.78	3.82	0.000	0.10	0.31	0.24
Psycho-somatic malaise	2.38	0.96	2.12	0.86	4.47	0.000	0.15	0.38	0.28
PTSS	1.44	0.97	1.02	0.92	6.68	0.000	0.30	0.54	0.44
CSE	5.44	1.40	5.66	1.31	−2.35	0.019	−0.39	−0.02	0.16
Job satisfaction	5.73	2.68	6.56	2.26	−5.29	0.000	−1.14	−0.52	0.33
Turnover intentions	3.11	2.07	2.57	1.90	4.12	0.000	0.28	0.80	0.27

**Note(s):** M = mean; SD = standard deviation; t = t-student value; *p* = *p* value; 95%CI = 95% confidence interval; LL = lower limit; UL = upper limit

**Source(s):** Authors' own work

1. **Profile 1: Moderate-High Mistreatment Exposure** (10.95%, *n* = 243) showed consistently elevated mistreatment across all dimensions. Physical aggression was confined mainly to this group. Members were predominantly female (81.3%) and nurses (55.4%). The mean age was 46.16 years (SD = 11.14). They reported extensive direct user contact (82.8% of working hours) and frequent shift work, including nights (81.9%).
2. **Profile 2: Low Mistreatment Exposure** (89.05%, *n* = 1976) consistently reported lower mistreatment, particularly physical aggression. This group was predominantly female (76.4%), with higher proportions of physicians (20.1%) and nurses (32.2%). Their mean age was 47.12 years (SD = 11.26), with 74.3% of hours spent in direct user contact and 67.1% of shifts worked, including nights (see Table 5 for details).

**Table 5:** Descriptive statistics of the two profiles (*N* = 2,219)

	Profile 1 (*n* = 243)	Profile 2 (*n* = 1976)
	%(*n*)	%(*n*)
*Gender*		
Women	81.30(196)	76.4(1499)
*Role*		
Doctor	14.6(35)	20.1(392)
Nurse	55.4(133)	32.2(629)
Healthcare assistants/auxiliary staff	13.0(31)	9.1(179)
Laboratory technician/radiologist	3.3(8)	8.6(168)
Other healthcare professions (e.g. psychologist, obstetrician, physiotherapist)	13.7(36)	70.0(608)
*Ward*		
Medicine	7.2(17)	4.6(89)
Surgery	5.1(12)	5.1(100)
Psychiatry	5.1(12)	1.5(29)
Emergency Room	16.1(38)	3.3(64)
Intensive care	1.7(4)	1.2(24)
Radiology	2.1(5)	3.4(67)
Cardiology	3.4(8)	3.2(63)
Haematology	2.5(6)	0.6(11)
Pulmonology	3.0(7)	2.8(56)
Orthopaedics	4.2(10)	3.7(73)
Paediatrics	2.5(6)	3.7(73)
Anaesthesia and Resuscitation	3.0(7)	2.1(41)
Gynaecology and Obstetrics	1.7(4)	2.6(52)
Others (e.g. infectious diseases, oncology, otolaryngology, neurosurgery, intensive care)	42.2(100)	58.6(1158)
*Night shifts*	81.9(140)	67.1(616)

	M(SD)	M(SD)
*Age*	46.16(11.14)	47.12(11.26)
*Direct contact with users*	82.89 (19.12)	74.25(24.35)

**Note(s):** Values represent percentages, with frequencies reported in parentheses. Age is expressed in years. Direct contact with users indicates the percentage of working time spent in direct contact with the public. M = mean; SD = standard deviation

**Source(s):** Authors' own work

**Figure A1:**
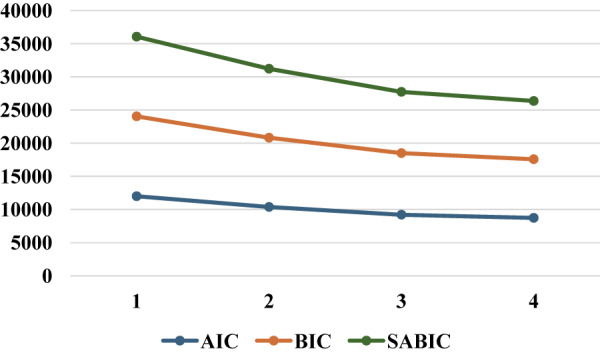
Elbow plot of fit indices from the latent profile analyses. **Source:** Authors' own work

Supplementary analyses confirmed the robustness of the two-profile solution. First, the elbows plot (Figure A1) showed stabilisation at two profiles. Next, Supplementary Table A1 showed that all retained profiles included at least 1% of participants. Finally, Supplementary Table A2 reported high average assignment probabilities (0.964–0.997) and minimal misclassification (0.003–0.036). Together, these supplementary checks further support the stability and reliability of the identified profiles.

### 3.4 Independent *t*-test analyses

Independent *t*-tests (Table 4) showed consistent differences between the two profiles across psychological and occupational well-being indicators. Healthcare professionals in the Moderate-High Mistreatment Exposure profile reported significantly higher emotional exhaustion (M = 2.90, SD = 1.01; *t*(2,215) = 6.85, *p* < 0.001), mental distance (M = 1.96, SD = 0.86; *t*(2,215) = 3.82, *p* < 0.001), psychosomatic symptoms (M = 2.38, SD = 0.96; *t*(2,215) = 4.47, *p* < 0.001), PTSS (M = 1.44, SD = 0.97; *t*(2,215) = 6.68, *p* < 0.001), and turnover intentions (M = 3.11, SD = 2.07; *t*(2,215) = 4.12, *p* < 0.001). They also reported lower CSE (M = 5.44, SD = 1.40; *t*(2,215) = −2.35, *p* = 0.019) and job satisfaction (M = 5.73, SD = 2.68; *t*(2,215) = −5.29, *p* < 0.001) than the Low Mistreatment Exposure group. Effect sizes indicated small yet meaningful differences for mental distance (d = 0.24), psychosomatic symptoms (d = 0.28), turnover intentions (d = 0.27), and CSE (d = 0.16), and moderate differences for emotional exhaustion (d = 0.46), PTSS (d = 0.44), and job satisfaction (d = 0.33). Overall, these findings indicate that cumulative exposure to outsider mistreatment is associated with markedly poorer psychological health and less favourable occupational attitudes.

## 4. Discussion

This study applied a person-centred approach to outsider mistreatment in healthcare, identifying two distinct exposure profiles: a Low Mistreatment group (89%) and a Moderate–High Mistreatment group (11%). The latter was characterised primarily by recurrent physical aggression, suggesting that for a minority of healthcare professionals, mistreatment is not episodic but persistent and severe. These findings are consistent and extend prior LPA studies of mistreatment in healthcare, which similarly revealed exposure patterns with meaningful differences in outcomes. For example, Pina *et al.* (2022) distinguished high- and low-violence profiles associated with differential levels of exhaustion, somatic complaints, and job satisfaction. In contrast, Chan *et al.* (2022) found that nurses with high exposure to bullying were more likely to report burnout and errors. Namin *et al.* (2022) also identified subgroups of frontline employees exposed to incivility, with greater exposure associated with greater exhaustion, poorer performance, and stronger turnover intentions. The present study extends this evidence by demonstrating that comparable latent patterns also characterise mistreatment originating from outsiders, rather than exclusively from insiders.

In line with the continuum of mistreatment model (Hershcovis, 2011; Townsley *et al.*, 2023), our results underscore that outsider incivility, verbal aggression, and physical aggression are interrelated expressions of interpersonal mistreatment rather than discrete phenomena. If left unaddressed, outsider incivility may escalate into more explicit hostility, consistent with the spiral of incivility (Andersson and Pearson, 1999). From a COR perspective (Hobfoll *et al.*, 2018), even subtle or low-intensity acts may gradually erode personal and occupational resources, increasing vulnerability to subsequent and more severe aggression. Thus, the two identified profiles can be interpreted as reflecting different positions along a cumulative mistreatment continuum shaped by resource loss processes.

Consistent with expectations, healthcare professionals in the Moderate–High Mistreatment profile reported significantly poorer psychological and occupational outcomes, including greater emotional exhaustion, psychosomatic symptoms, mental distancing, and PTSSs, as well as lower job satisfaction and stronger turnover intentions. Rather than reiterating individual associations, these findings collectively support the notion that sustained mistreatment undermines both well-being and work-related attitudes through cumulative loss processes, in line with previous person-centred research (Chan *et al.*, 2022; Pina *et al.*, 2022; Namin *et al.*, 2022). Notably, the findings highlight the traumatic potential of outsider mistreatment. Professionals in the Moderate–High profile reported elevated PTSSs, including intrusion, avoidance, and hyperarousal. Even at subclinical levels, such symptoms are known to impair attention, decision-making, and emotional regulation (Hou *et al.*, 2024; Montani *et al.*, 2020; Sommovigo *et al.*, 2025a, b), thereby compounding resource loss. Moreover, members of this profile reported significantly lower trauma-related coping self-efficacy (CSE), suggesting that persistent exposure to aggression may erode (rather than strengthen) beliefs in one's capacity to cope effectively (Sommovigo *et al.*, 2025a, b), thereby weakening a key internal resource within COR loss spirals. This pattern is consistent with COR's desperation principle (Hobfoll *et al.*, 2018), which holds that severe and sustained losses compromise individuals' ability to conserve or replenish resources. At the same time, the negative associations between CSE and strain outcomes reinforce its role as a key protective resource supporting adaptive appraisal and coping under conditions of threat (Bosmans and van der Velden, 2017; Luszczynska *et al.*, 2009).

Beyond psychological and attitudinal outcomes, profile membership was associated with specific job characteristics. The Moderate–High profile included a higher proportion of nurses, night-shift workers, and staff with extensive direct contact with service users—conditions that combine intense relational demands with constrained structural protection, heightening the risk of mistreatment (Baek and Trinkoff, 2022; Chen *et al.*, 2024). Demographic patterns also suggested greater vulnerability among younger and female professionals, consistent with prior findings highlighting power and authority dynamics in exposure to mistreatment (Cortina *et al.*, 2017; Layne *et al.*, 2019; Sommovigo *et al.*, 2022). Taken together, these results reinforce the conceptualisation of outsider mistreatment as a cumulative, resource-driven process with heterogeneous manifestations and consequences. By incorporating CSE into a person-centred framework, this study adds nuance to the literature, showing that mistreatment not only depletes emotional and physical resources but can also undermine core coping beliefs—thereby perpetuating cycles of vulnerability.

### 4.1 Theoretical implications

This study makes several substantive theoretical contributions to the literature on workplace mistreatment and aggression in healthcare. Adopting a person-centred latent profile approach, it moves beyond dominant variable-centred models that implicitly assume homogeneous exposure and effects, demonstrating that outsider mistreatment is structured into qualitatively distinct exposure configurations with differential implications for resource depletion, coping capacity, and work-related functioning. Drawing on data from healthcare professionals employed across eight public hospitals, the study extends prior person-centred research that has primarily relied on single-organisation or small-sample designs (Kusurkar *et al.*, 2021; Morin *et al.*, 2018). Focusing on public hospitals is theoretically consequential, as these organisations are characterised by high service demand, constrained resources, and limited discretion over managing patient flows, all of which amplify structural exposure to outsider mistreatment while simultaneously restricting opportunities for resource replenishment (Lim *et al.*, 2022; O'Brien *et al.*, 2024; Mento *et al*., 2020). This multi-organisational perspective enhances the external validity of the identified profiles. It provides evidence that heterogeneous patterns of outsider mistreatment are not context-specific anomalies but rather reflect broader, systemic dynamics of interaction and vulnerability embedded within public healthcare systems, where professionals are required to deliver care under conditions of chronic workload, normative expectations of emotional labour, and reduced organisational buffering against aggression (NIOSH, 2022; Townsley *et al.*, 2023; Mento *et al.*, 2020). In doing so, the study advances person-centred and resource-based perspectives by conceptualising outsider mistreatment as a differentiated stress process with unequal consequences across subgroups, thereby extending occupational aggression research beyond homogeneous, one-size-fits-all conceptualisations. Second, the findings extend prior person-centred research on workplace mistreatment, which has predominantly focused on internal sources such as bullying and lateral violence (Baek and Trinkoff, 2022; Chan *et al.*, 2022), by demonstrating that comparable heterogeneity also characterises mistreatment originating from outsiders. Whereas existing latent profile studies have primarily examined aggression embedded within intra-organisational relationships, the present study shows that exposure to mistreatment initiated by patients, relatives, and visitors similarly clusters into distinct configurations with differential implications for employee functioning. This contribution broadens the conceptual boundaries of mistreatment research and responds to calls to more fully integrate external sources of aggression into organisational stress and occupational health frameworks (Cheng *et al.*, 2020; Townsley *et al.*, 2023), highlighting that the perpetrator's “outsider” status does not diminish the systematic nature of the exposure patterns. Third, this study is the first to demonstrate that distinct profiles of exposure to outsider aggression are systematically and differentially associated with trauma-related coping self-efficacy. Whereas prior research has primarily focused on well-being, burnout, or attitudinal outcomes (Mawuena *et al.*, 2024; Mento *et al.*, 2020), the present findings show that recurrent exposure to incivility, verbal aggression, and especially physical aggression is also linked to a diminished perceived capacity to cope with potentially traumatic events (Benight and Bandura, 2004; Sommovigo *et al.*, 2025a, b). By responding to recent calls to examine the impact of outsider aggression on domain-specific forms of self-efficacy among healthcare professionals (Cavallari *et al.*, 2025a, b), the study extends COR theory (Hobfoll *et al.*, 2018) by showing that repeated mistreatment not only depletes emotional and physical resources but also erodes core coping beliefs, thereby reinforcing loss spirals. These beliefs are central to individuals' ability to regulate distress, mobilise adaptive coping strategies, and sustain functioning under conditions of threat. This finding helps explain how cumulative exposure to aggression translates into heightened vulnerability over time, as the very internal mechanisms intended to protect against stress are themselves compromised by the stressor (Luszczynska *et al.*, 2009; Sommovigo *et al.*, 2025a, b). Finally, by explicitly linking profiles of mistreatment exposure to CSE and PTSS, this study further advances the conceptualisation of workplace aggression as a potentially traumatic occupational stressor (Dai *et al.*, 2024; Sommovigo *et al.*, 2025a; Wagner *et al.*, 2024), rather than merely a source of temporary strain or job dissatisfaction. The findings indicate that incivility, verbal aggression, and physical aggression co-occur as cumulative stressors along a continuum of mistreatment (Hershcovis, 2011; Townsley *et al.*, 2023), where even low-intensity acts of incivility may act as precursors to more severe resource depletion (Andersson and Pearson, 1999; Chris *et al.*, 2022; Thomas *et al.*, 2022). Moreover, persistent aggression from outsiders appears to shape how healthcare professionals appraise both future adverse events and their own capacity to manage them, thereby fostering cumulative vulnerability and resource loss over time. Taken together, these contributions refine the theoretical understanding of outsider mistreatment as a resource-based and potentially traumatic phenomenon characterised by heterogeneous exposure patterns and unequal vulnerability, with implications that extend beyond traditional models of occupational stress and underscore the value of integrating trauma-informed frameworks into the study of workplace aggression.

### 4.2 Limitations and future research directions

This study adopts a person-centred approach to elucidate the heterogeneous ways in which healthcare professionals experience outsider mistreatment. By identifying distinct exposure profiles and linking them to well-being and work-related outcomes, the findings underscore the complexity of mistreatment and highlight subgroups most in need of tailored interventions. However, some limitations should be acknowledged. First, the cross-sectional design limits causal inference; longitudinal studies are needed to clarify temporal dynamics and potential shifts in profile membership. Second, reliance on self-report data may introduce bias, despite tests suggesting minimal risk of common method bias. Future research would benefit from integrating multi-source assessments (e.g. peer- and supervisor-reported data, incident reports) and objective indicators (e.g. absenteeism, turnover). Third, although data were collected from eight hospitals in Northern Italy, generalisability is limited; replication across diverse cultural and organisational contexts, including private healthcare systems, is warranted. Fourth, the two-profile solution, though parsimonious and robust, may not fully capture the spectrum of mistreatment experiences. Studies with larger and more diverse samples could uncover additional subtypes. Finally, future work should examine moderating and contextual factors (e.g. leadership climate, peer support, emotion regulation), long-term consequences (e.g. career trajectories, burnout recovery), and the combined effects of external and internal sources of mistreatment. Incorporating these dimensions into latent profile analyses could yield a more comprehensive understanding of risk and resilience and support the design of context-sensitive interventions that address subgroup-specific needs.

### 4.3 Practical implications

Systematic monitoring of mistreatment by outsiders can enable healthcare organisations to identify distinct exposure profiles and implement targeted, evidence-based interventions rather than relying on uniform, one-size-fits-all policies. Our findings show that professionals in the Moderate–High Exposure profile experience substantially poorer psychological and occupational well-being than those in the Low Exposure profile. These differences underscore the limitations of generic prevention approaches and highlight the need for early identification and differentiated support pathways. Organisations could translate these findings into practice by embedding routine screening for exposure to mistreatment within occupational health surveillance or staff well-being dashboards (e.g. periodic brief checklists integrated into staff surveys or incident-reporting systems). Healthcare professionals identified as belonging to higher-risk profiles could then be proactively referred to tiered intervention programmes, such as post-incident debriefings following aggressive events, access to short-term psychological counselling, or trauma-informed support pathways (Al Smadi, 2025). Training initiatives focusing on resilience, recovery following exposure, and the development of supervisors' empathetic leadership skills may be particularly beneficial for these groups (Cavallari *et al.*, 2025b; Lim *et al.*, 2022).

By identifying distinct aggression profiles, healthcare organisations can strategically allocate preventive resources. This approach enables more effective use of often-limited resources by prioritising interventions for groups at higher risk and greater vulnerability. In the present study, the Moderate–High profile was disproportionately associated with nursing roles, extensive patient contact, and shift work, especially night shifts. Organisations could respond by implementing role- and schedule-sensitive interventions, such as emotion-regulation training (e.g. mindfulness-based practices, cognitive restructuring exercises) delivered within continuing professional development programmes, formal mentoring systems for early-career nurses, or enhanced supervisory presence during high-risk shifts. Where feasible, flexible scheduling, job rotation, or temporary redeployment away from high-exposure units may help reduce cumulative risk while supporting retention. Given that physical aggression was almost exclusively concentrated in the Moderate-High profile, safety-oriented measures remain essential for this group. Practical actions include environmental redesign (e.g. improved visibility and secure layout), enhanced security presence in high-risk units, transparent and accessible reporting procedures, and regular de-escalation training tailored to frontline staff. Moreover, the lower trauma-related CSE observed in this group suggests the value of Coping Effectiveness Training, which integrates cognitive restructuring, relaxation, and problem-solving techniques to bolster internal resources (Benight and Bandura, 2004; Bosmans and van der Velden, 2017). At the broader organisational level, embedding formal anti-aggression protocols, standardised reporting guidelines, and a culture that actively de-normalises mistreatment is critical. Team-level practices, including structured peer debriefings, facilitated reflective sessions, and psychologically safe spaces for discussing incidents of aggression, may further enhance collective resilience and cohesion. While high-risk profiles require immediate prioritisation, extending lighter preventive measures (e.g. awareness training, early warning indicators) to low-exposure groups can support early detection of emerging risks and prevent escalation over time.

Beyond employee well-being, identifying distinct exposure profiles has substantial economic, operational, and policy implications for healthcare organisations. Exposure to mistreatment by outsiders is a well-documented driver of absenteeism, presenteeism, increased turnover intentions, and reduced productivity, all of which translate into high direct and indirect organisational costs. From a cost-containment perspective, early identification of professionals in Moderate–High exposure profiles enables healthcare organisations to allocate preventive resources more strategically, thereby maximising the effectiveness of often-limited economic investments. By targeting interventions towards higher-risk and more vulnerable groups, organisations may reduce avoidable turnover, retain experienced staff, and limit downstream costs associated with sickness absence, replacement, retraining, and productivity losses, as well as potential costs related to compromised patient safety. In this sense, investments in prevention and tailored support should be understood not only as ethical imperatives but also as economically sustainable workforce strategies capable of generating a measurable return on investment (ROI) in occupational health.

From an organisational and operational standpoint, workforce stability is a critical performance asset, even within public or quasi-market healthcare systems. Work environments perceived as safe and supportive enhance employer attractiveness and retention capacity. They also promote continuity of care in increasingly competitive and resource-constrained labour markets. Reducing cumulative exposure to aggression and staff distress may also lower the risk of clinical errors and adverse events. This, in turn, mitigates downstream costs associated with litigation, insurance coverage, and service disruptions. Profile-based intervention strategies contribute not only to employee protection but also to service quality, organisational resilience, and the sustainable delivery of care.

The findings are equally relevant from a public policy perspective, particularly in the context of large-scale workforce and health system reform initiatives, such as those promoted under the Italian National Recovery and Resilience Plan (PNRR) and the broader Next Generation EU framework. In publicly funded healthcare systems, chronic workforce strain, burnout, and turnover generate cascading inefficiencies that threaten the long-term sustainability of the system. Evidence from differentiated exposure profiles can support policymakers in developing data-informed occupational health regulations, national guidelines on violence prevention, and targeted workforce protection strategies. Notably, a stratified, profile-based prevention model enables a more efficient allocation of public resources by prioritising funding and preventive efforts for units, roles, or working conditions characterised by higher risk (e.g. frontline nursing roles or high-intensity clinical settings), rather than relying on generic, low-impact interventions. In summary, linking patterns of outsider mistreatment to both employee well-being and organisational performance highlights the value of a differentiated, data-driven prevention approach. Such a strategy offers dual returns. It safeguards employee health and retention, improves organisational efficiency, and supports public policy objectives aimed at sustaining high-quality, resilient healthcare systems.

## 5. Conclusions

This study adopts a person-centred approach to outsider mistreatment in healthcare, highlighting how heterogeneous exposure patterns shape psychological and occupational outcomes. Two distinct profiles emerged: a predominant Low Mistreatment group and a smaller yet significant Moderate–High Mistreatment group. The latter was characterised by elevated incivility, verbal aggression, and physical aggression, and was associated with greater emotional exhaustion, psychosomatic complaints, mental distancing, and PTSSs, as well as lower job satisfaction, reduced trauma-related CSE, and stronger turnover intentions. These findings underscore that outsider mistreatment cannot be addressed through one-size-fits-all approaches. Healthcare organisations must implement differentiated, evidence-based strategies that address the specific vulnerabilities of high-exposure groups while sustaining preventive measures for the broader workforce. Tailored interventions can protect well-being, strengthen coping resources, and ultimately support workforce stability and quality of care.

## Supplementary Materials

Table A1 shows profile membership for the 2 to 4-profile solutions. Table A2 shows that the average probability of individuals being correctly assigned to their profile was high (0.997–0.964), whereas probabilities for other profiles were much lower (0.003–0.036).

**Table A1:** Profile membership across alternative latent profile solutions

Latent profile	%(*n*)			
n-profile	1	2	3	4
2-profile	89.05(1976)	10.95(243)		
3-profile	87.56(1943)	8.24(183)	4.19(93)	
4-profile	87.47(1941)	0.45(10)	8.34(185)	3.74(83)

**Note(s):** Values represent percentages, with frequencies reported in parentheses

**Source(s):** Authors' own work

**Table A2:** Average latent class probabilities for most likely latent class membership (Row) by latent class (Column)

	1	2
1	*0.964*	0.036
2	0.003	*0.997*

**Source(s):** Authors' own work

Figure A1 shows the elbow plot of these indices, indicating that a plateau was reached at the two-profile solution, supporting the choice of the two-profile solution.
